# Breastfeeding patterns and factors associated with early weaning in the Western Amazon

**DOI:** 10.11606/s1518-8787.2021055002134

**Published:** 2021-05-06

**Authors:** Fernanda Andrade Martins, Alanderson Alves Ramalho, Andréia Moreira de Andrade, Simone Perufo Opitz, Rosalina Jorge Koifman, Ilce Ferreira da Silva

**Affiliations:** I Universidade Federal do Acre Programa de Pós-Graduação em Saúde Coletiva Centro de Ciências da Saúde e do Desporto Rio BrancoAC Brasil Universidade Federal do Acre. Centro de Ciências da Saúde e do Desporto. Programa de Pós-Graduação em Saúde Coletiva. Rio Branco, AC, Brasil.; II Fundação Oswaldo Cruz Escola Nacional de Saúde Pública Sérgio Auroca Departamento de Epidemiologia e Métodos Quantitativos em Saúde Rio de JaneiroRJ Brasil Fundação Oswaldo Cruz. Escola Nacional de Saúde Pública Sérgio Auroca. Departamento de Epidemiologia e Métodos Quantitativos em Saúde. Rio de Janeiro, RJ, Brasil.

**Keywords:** Breast Feeding, Weaning, Infant Nutrition, Child Health, Health Status Indicators

## Abstract

**OBJECTIVE:**

To characterize breastfeeding patterns in the first six months of life and factors associated with early weaning in a birth-cohort in Rio Branco, state of Acre.

**METHODS:**

This is a prospective study with all babies born between April and June 2015. The mothers were interviewed soon after birth and between 6 and 15 months postpartum. At hospital discharge, breastfeeding was defined as exclusively (EBF), and breastfeeding (BF). In the follow-up, breastfeeding patterns were exclusive breastfeeding (EBF), predominant breastfeeding (PBF), and breastfeeding (BF). The interruption of breastfeeding in the first six months was classified as early weaning. The Kaplan Meier method (log-rank: 95%) was used to estimate the conditional probability of change in breastfeeding pattern, and early weaning risk. Crude and adjusted proportional Cox regression models, and their respective 95% confidence intervals (95%CI), were used to analyze the factors associated with early weaning.

**RESULTS:**

The study included 833 infants in EBF (95.4%) and BF (4.6%) at hospital discharge. During the first six months of life, the infant likely discharged in EBF remaining in EBF, becoming PBF, and BF, were respectively 16.4%, 32.3%, and 56.5%. The weaning likely at six months was statistically higher for infants discharged in BF (47.4%) when compared with those discharged in EBF (26%). Factors associated with early weaning were BF at hospital discharge (HR = 1.82; 95%CI 1.06–3.11), no mother cross-breastfeeding (HR = 2.50; 95%CI 1.59–3.94), pacifier use (HR = 6.23; 95%CI 4.52–8.60), less than six months of breastfeeding intention (HR = 1.93; 95%CI 1.25–2.98), lack of breastfeeding in the first hour of life (HR = 1.45; 95%CI 1.10–1.92), and pregnancy alcohol consumption (HR = 1.88; 95%CI 1.34–2.90).

**CONCLUSION:**

Compared to infants in EBF, those in BF at hospital discharge were more likely to wean. Public health efforts should prioritize EBF at hospital discharge, promote breastfeeding in the first hour of life, and prevent alcohol consumption risks during pregnancy, cross-breastfeeding and pacifier use.

## INTRODUCTION

Exclusive breastfeeding up to six months of age is one of the main objectives of nutrition and public health programs to reduce child mortality under 5-years old^1^. The short and long-term breastfeeding benefits for child-and-mother health are widely recognized^1^. Therefore, the World Health Organization (WHO)^2^ recommends that such benefits should be improved when combining exclusive breastfeeding during the first months and complemented breastfeeding for at least two years. However, many women quit breastfeeding before the recommended timing^3^^–^^7^.

National surveys conducted since 1975 in Brazil have shown an increase in exclusive breastfeeding (EBF) in children between 0 and 6 months, and an increase in the median duration of breastfeeding, reaching WHO’s recommendations^8^. Such an achievement is probably related to public health actions undertaken in the last 30 years promoting breastfeeding. Trend series of breastfeeding (BF) indicators in Brazil showed an increasing trend until 2006, followed by stabilized trends for the BF in children under six months, the continued breastfeeding with one year of life, and the breastfeeding in children under 2-years old. Such results point out the need to evaluate and revise breastfeeding promotion, protection, and support policies and programs in the country^9^. In 2008, the Survey on the Breastfeeding Prevalence in all Brazilian state capitals and the Federal District, observed that in Rio Branco, capital of the state of Acre, the prevalence of EBF in children under six months was 36.1%, which is lower than the national average in the same year (41%)^10^.

Factors frequently associated with BF discontinuity include first pregnancy^7^^,^^11^^–^^13^, low birth weight^3^^,^^12^^,^^14^, pacifier use^3^^,^^7^^,^^12^, maternal difficulty to breastfeed after childbirth^4^^,^^11^^,^^15^, late onset of BF^3^, lack of EBF in maternity^4^^,^^11^^,^^14^, maternal misunderstanding related to ideal BF timing under six months^11^, lack of breastfeeding advantages awarness^11^, lack of breastfeeding paternal support^7^^,^^11^, working mothers^12^^–^^14^, tobacco, and alcohol use^3^^,^^15^, maternal young age^12^^,^^13^^,^^15^, and maternal education^3^^,^^4^^,^^12^^,^^13^^,^^15^.

The maternal intentions and confidence related to breastfeeding, and family support to avoid maternal isolation in the puerperium could help BF continuity, whereas anxiety and inexperience to deal with the motherhood have generated a reverse effect^3^^,^^4^^,^^13^^,^^15^^,^^16^. In this sense, determining the breastfeeding profile and early weaning associated factors is important to support culturally adjusted breastfeeding policies development. Thus, this study aimed to characterize breastfeeding in the first six months patterns and early weaning associated factors in a birth-cohort in Rio Branco, state of Acre.

## METHODS

This study is part of the project “Evolution of nutritional indicators of children from birth to the first year of life in Rio Branco, Acre,” designed to investigate child health from birth up to two years old in the only two maternity hospitals in the capital. The sample size estimation of the main project was based on the number of deliveries in 2010 of women living in Rio Branco (6,437)^17^. Considering the frequency of “breastfeeding in the first hour of life” as exposure, a type-I error of 5%, power of 80%, exposed/unexposed ratio of 9, and risk/prevalence ratio of 2.5, it was established that it would be necessary to include in the sample 1,192 mother-child binomial.

All participants formalized their participation in the study by signing the informed consent form. The present study was approved by the Research Ethics Committee of the *Universidade Federal do Acre.* 40584115.0.0000.5010.

This is a prospective study in a birth-Cohort of Rio Branco, State of Acre, between April 6 and June 30, 2015, and followed between the 6th and 15th postpartum month. Inclusion criteria encompassed the infants whose mothers lived in the urban area of Rio Branco and did not present diagnosed psychiatric disorders keeping them from answering the interview. In the present study, the exclusion criteria included twins, infants who presented breastfeeding contraindication, and those who were never breastfed or breastfed for less than one day.

Data were collected using standardized instruments, applied by a team of health students trained and supervised by researchers from the Universidade Federal do Acre, responsible for fieldwork quality control. The first interview was conducted in the first 48 hours postpartum at the hospital, and the follow-up visit was scheduled by telephone. When telephone contact was not possible, a home visit was performed at the previously informed address. At the time of the home visit, whether the participant was not at home, new home-visits were proceeded at an alternate schedule, including weekends (an average of three attempt-visits).

The independent variables were obtained by interview and confirmed directly from medical records, the birth certificate, and the pregnancy card report. Variables included maternal age (<20, 20–34 or ≥ 35 years); skin color; maternal education (some or middle school, some or high school, some or higher education); marital status (without or with a partner); number of child’s siblings (none, 1– 2 or 3 or more); smoking during pregnancy (yes or no); prenatal care sector (public or private); the number of prenatal appointments (≤ 5, 6 or > 6 appointments); delivery type (natural or cesarean section); premature birth (yes or no); low birth weight (yes or no); and baby gender (male or female).

The variables obtained exclusively by the interview proceeded in the maternity included beneficiary of income transfer program (Bolsa Família) (yes or no); planned pregnancy (yes or no); alcohol consumption during pregnancy (yes or no); breastfeeding in the first hour of life (yes or no); complemented breastfeeding in the hospital (yes or no); professional assistance in BF procedures (yes or no); intended breastfeeding timing (< 6, 6 or > 6 months); possession items (consumer goods); the head-family education (up to middle school, complete/incomplete high-school, complete/incomplete college).

Socioeconomic status was defined according to criteria of the *Associação Brasileira de Empresas de Pesquisa* (Brazilian Association of Research Companies)^18^, and categorized into classes A and B or C, D and E. The variable “maternal skin-color” was self-declared, obtained according to the classification of the Brazilian Institute of Geography and Statistics, and categorized as “white,” “brown/mixed race”, and “others”^19^. Maternal education was collected categorically, which made impossible its analysis by year of schooling. Low birth weight included babies with lower than 2,500 g weight^20^, and premature babies included those born with less than 37 weeks of gestational age^21^.

In the follow-up interview, information on infant diet was obtained. The frequencies of breastfeeding practices were estimated at hospital discharge and follow-up, based on the WHO definition^22^. Children fed with breast milk only were classified as EBF, and those who received breast milk, and other milk-kind, were classified as BF. During follow-up, the breastfeeding pattern for children in BF at discharge was defined as predominant breastfeeding (PBF) when breast milk was associated with water, teas, or fruit juice, and BF when breastfeeding was associated with other milk-kind, or any solid or semisolid food.

Early weaning was defined as the interruption of BF in the first six months of life. Time to weaning was measured as the days between the date of birth and BF interruption. Infants in EBF, PBF, or BF at six months were censored in the cohort.

The breastfeeding time (days) for each breastfeeding pattern (EBF, PBF, or BF) was created based on the age of introduction of water, tea, or juice (days); other milk introduction age (days); and age of introduction of other foods (days). The variables early weaning (yes or no) and breastfeeding time until weaning (days) were based on breastfeeding interruption (days). The mothers were also asked if they quit working after birth delivery (yes or no), if they cross-breastfed other women’s infants (yes or no), if the infant received breast milk from another mother (cross-breastfed babies: yes or no), or if they used pacifiers (pacifier use: yes or no) if the child’s father participation was positive in encouraging breastfeeding (yes or no), and the reasons for complemented breastfeeding in the hospital.

The participants’ characteristics were described using means (standard deviation) for continuous variables, and proportions (%) for categorical variables. The Pearson chi-square test or Fisher test was used to comparing the characteristics of the participants included in the analyses and those lost in the follow-up.

The conditional probabilities for changing the breastfeeding pattern at 30, 60, 90, 120, and 180 days, the risk of weaning according to the breastfeeding patterns at hospital discharge, the sociodemographic variables, maternal habits, prenatal and hospital care, and the characteristics of the child were estimated by the Kaplan-Meier method. The differences between the curves were evaluated by the log-rank test (95%).

Crude and adjusted hazard ratios (HR), and their respective 95% confidence intervals (95%CI), for early weaning were estimated by the proportional Cox regression model. In the adjusted analysis, we considered as an entry-criteria in the model a p-value < 0.20 in the crude analyses and biological importance to the outcome. Variables presenting a p-value of ≤ 0.05 in the model or biological importance were maintained in the final model. The maximum likelihood log was used to estimate the coefficients of the model. The global and partial R² was used to evaluate the goodness-of-fit models, while log minus log curves were used to test the assumption of proportional risks over time. The statistical analyses were proceeded using the Statistical Package for the Social Sciences, SPSS, version 22.0.

## RESULTS

**Figure 1:**
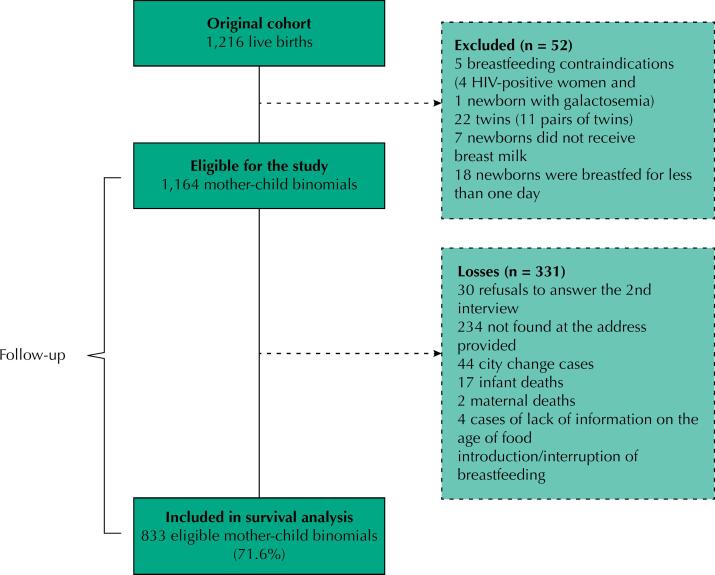
Flowchart of participants in the birth cohort “Evolution of nutritional indicators of children from birth to first year of life in Rio Branco, Acre” eligible for the study.

From 1,216 LB in the original cohort, 52 (4.3%) infants were excluded due to breastfeeding contraindications (n = 5), were twin (n = 22), were not breastfed (n = 7) or were breastfed for less than one day (n = 18), leaving 1,164 infants eligible for our study. During follow-up, 331 (28.4%) participants were lost for refusing to answer the second interview (n = 30), because they were not found at the provided address (n = 234), moving to another city (n = 44), maternal death (n = 2), child death (n = 17) or lack of the outcome information (n = 4). Thus, the study population consisted of 833 infants, representing 71.6% of the eligible binomials (Figure 1). In the analyses, the follow-up losses were compared with the included participants, and there were no statistical differences for all sociodemographic characteristics.

The mothers participating in the study presented a mean age of 25.23 years (± 6.8, data not presented), and 26.3% were under 20 years of age, 21.7% had studied up to middle school (complete or incomplete), 18.8% were beneficiaries of the Brazilian *income-transfer* program, and 83.4% self-declared as brown skin color. Moreover, 69.4% attended at least six prenatal appointments, and 85.1% attended prenatal care in the public sector. Among the infants, 51.3% were girls, 49% were born by cesarean section, 7.5% had low birth weight, and 58% started breastfeeding in the first hour of life. At hospital discharge, 95.4% of the infants were in EBF, and 4.6% in BF. However, 15% of the infants received complemented breastfeeding during still in the hospital (Table 1).

**Table 1:** Characteristics of the mother-child binomials participating in the birth cohort “Evolution of nutritional indicators of children from birth to the first year of life in Rio Branco, Acre.

Variable (n)^a^	Category	n (%)
Maternal age (years)		
	≥ 35	95 (11.4)
	20-34	519 (62.3)
	< 20	219 (26.3)
Maternal skin color		
	Brown/Mixed race	694 (83.4)
	White	82 (9.9)
	Others	56 (6.37)
Maternal education^b^		
	Up to middle school	203 (24.4)
	High school	438 (52.6)
	Higher education	192 (23.0)
Marital status		
	With partner	706 (84.8)
	Without partner	127 (15.2)
Working mother before pregnancy		
	Yes	285 (36.0)
	No	507 (64.0)
Quit working after birth delivery		
	No	323 (46.3)
	Yes	375 (53.7)
Socioeconomic status		
	A and B	170 (20.6)
	C, D and E	655 (79.4)
Beneficiary of income transfer program (Bolsa Família)		
	No	639 (81.2)
	Yes	148 (18.8)
Number of child’s siblings		
	3 or more	244 (29.4)
	1 or 2	251 (30.2)
	None	335 (40.4)
Planned pregnancy		
	Yes	530 (63.9)
	No	300 (36.1)
Smoking in pregnancy		
	No	761 (91.4)
	Yes	72 (91.34)
Alcohol consumption during pregnancy		
	No	726 (87.9)
	Yes	100 (12.1)
Prenatal care sector		
	Public	690 (85.1)
	Private	121 (14.9)
Number of prenatal appointments		
	≤ 5	252 (30.5)
	6	138 (16.7)
	> 6	435 (52.7)
Type of delivery		
	Normal	425 (51.0)
	Cesarean section	408 (49.0)
Baby gender		
	Boy	406 (48.7)
	Girl	427 (51.3)
Prematurity		
	No	762 (92.0)
	Yes	66 (8.0)
Low birth weight		
	No	767 (92.5)
	Yes	62 (7.5)
Breastfeeding in the first hour of life		
	Yes	471 (58.0)
	No	341 (42.0)
Hospital assistance in the management of breastfeeding		
	No	398 (48.2)
	Yes	427 (51.8)
Intended breastfeeding timing		
	> 6 months	399 (48.8)
	6 months	339 (41.5)
	< 6 months	79 (9.7)
Cross-breastfed babies		
	No	688 (82.6)
	Yes	145 (17.4)
Cross-breastfed other women’s infants		
	Yes	155 (18.6)
	No	678 (81.4)
Pacifier use		
	No	494 (59.5)
	Yes	336 (40.5)
Positive paternal participation in breastfeeding		
	Yes	681 (82.0)
	No	149 (18.0)
Complementation to breastfeeding in the hospital		
	Yes	125 (15.0)
	No	708 (85.0)
Breastfeeding status at discharge		
	EBF	795 (95.4)
	BF	38 (4.6)

BF: breastfeeding; EBF: exclusive breastfeeding.

a The differences when compared with the total are due to lack of information in the variable.

b The categories refer to the level of education.

The reasons for hospital complemented breastfeeding reported by the mothers included conditions related to the infant, such as premature babies, pathology or hypoglycemia (54.7%), mothers with low breast milk production or child with suction difficulty (35.8%), hospital routine (6.3%), and maternal medication use (3.2%). Thirty mothers were unable to provide the reason (data not presented).

**Figure 2:**
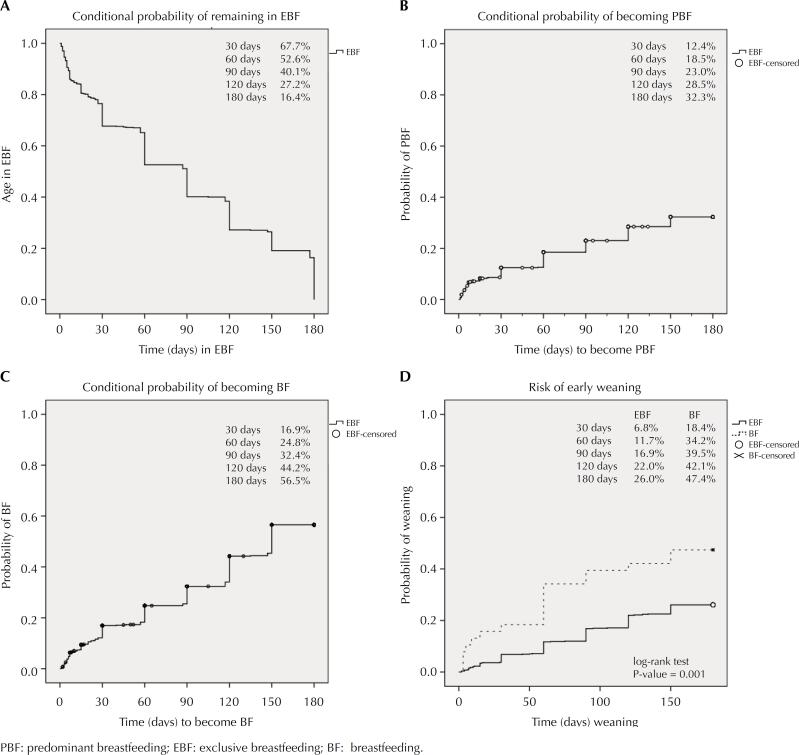
Breastfeeding patterns in the first six months of life (graphs A, B and C) and risk of early weaning (graph D), according to breastfeeding at hospital discharge, in the cohort of live births in Rio Branco, Acre (Kaplan Meier method).

BF median days were 180, and EBF mediandays were 90 days. BF mean duration were 152.53 days (± 51.25), and the EBF was 86.84 days (± 62.62). The conditional probability of weaning in the whole sample was 27% (data not presented). An EBF discharged child was 16.4% likely to remain exclusively breastfed, 32.3% likely to become PBF, and 56.5% to become BF in six months. In the hospital discharge, infants in BF (47.4%) were 26% more likely to early weaning than those discharged in EBF (Figure 2).

The statistically associated factors to early weaning in the crude analysis were alcohol consumption during pregnancy, absence of breastfeeding in the first hour of life, professional assistance in BF management, intention to breastfeed less than six months, BF at hospital discharge, no maternal cross-breastfeding other women’s infants, pacifier use, and the lack of positive paternal participation in BF (Table 2).

**Table 2:** Weaning in the first six months of life, according to sociodemographic variables, maternal habits during pregnancy, prenatal and hospital care and characteristics of the child. Rio Branco, Acre, 2015–2016.

Variable		Kaplan-Meier Analysis		Hazard Ratio (HR)
		1-Survival	log-rank p-value	crude HR [95%CI]
Maternal age			0.225	
	≥ 35 years	22.1		1
	20-34 years old	28.9		1.19 (0.72–1.97)
	< 20 years	24.7		1.41(0.90–2.22)
	Maternal skin color		0.285	
	Brown/Mixed race	26.1		1
	White	34.1		1.36 (0.91–2.03)
	Others	28.6		1.13 (0.68–1.89)
Maternal Education^a^			0.134	
	Up to middle school	21.7		1
	High school	29.7		1.40 (1.00–1.97)
	Higher education	26.6		1.23 (0.82–1.84)
Marital status			0.307	
	With partner	26.5		1
	Without partner	29.9		1.20 (0.84–1.70)
Working mother before pregnancy			0.417	
	Yes	28.8		1
	No	25.6		0.90 (0.68–1.18)
Quit working after birth delivery			0.198	
	No	23.8		1
	Yes	28.5		1.21 (0.90–1.62)
Socioeconomic status			0.694	
	A and B	28.2		1
	C, D and E	26.6		0.94 (0.69–1.30)
Beneficiary of income transfer program (Bolsa Família)			0.827	
	No	28.0		1
	Yes	26.4		0.97 (0.70–1.37)
Number of child’s siblings			0.180	
	3 or more	23.0		1
	1 or 2	27.5		1.23 (0.87–1.75)
	None	29.6		1.35 (0.98–1.88)
Planned pregnancy			0.063	
	Yes	23.9		1
	No	29.1		1.30 (0.98–1.72)
Smoking in pregnancy			0.054	
	No	26.3		1
	Yes	34.7		1.50 (0.99–2.26)
Alcohol consumption during pregnancy			0.005	
	No	25.6		1
	Yes	37.0		1.62 (1.14–2.31)
Prenatal care sector			0.232	
	Public	25.7		1
	Private	31.4		1.23 (0.87–1.75)
Number of prenatal appointments				
	≤ 5	25.8	0.958	1
	6	26.8		0.05 (0.70–1.57)
	> 6	27.6		1.04 (0.77–1.40)
Type of delivery			0.406	
	Normal	25.6		1
	Cesarean section	28.4		0.90 (0.70–1.16)
Prematurity			0.454	
	No	27.4		1
	Yes	22.7		0.82 (0.48–1.39)
Low birth weight			0.109	
	No	26.3		1
	Yes	35.5		1.42 (0.91–2.20)
Breastfeeding in the first hour of life			0.016	
	Yes	23.8		1
	No	31.1		1.38 (1.05–1.80)
Hospital assistance in the management of breastfeeding			0.027	
	No	23.9		1
	Yes	30.2		1.34 (1.03–1.75)
Intended breastfeeding timing			0.012	
	6 months	22.8		1
	6 months	28.6		1.29 (0.98–1.72)
	< 6 months	38.0		1.80 (1.19–2.71)
Breastfeeding status at discharge			0.001	
	EBF	26.0		1
	BF	47.4		2.24 (1.38–3.63)
Cross-breastfed babies			0.078	
	No	28.3		1
	Yes	20.7		0.71 (0.48–1.05)
Cross-breastfed other women’s infants			p < 0.001	
	Yes	13.5		1
	No	30.1		2.46 (1.57–3.86)
Pacifier use			p < 0.001	
	No	10.1		1
	Yes	51.2		6.28 (4.60–8.57)
Positive paternal participation in breastfeeding			0.009	
	Yes	25.3		1
	No	34.2		1.50 (1.10–2.05)

a The categories refer to the level of education.

In the multiple analysis, the BF at hospital discharge increased by 82% of the early weaning risk compared with the EBF. This risk was also 45% higher in infants not breastfed in the first hour of life. Infants that used pacifiers had a 6.23 times higher risk of early weaning than those that did not use pacifiers. The risk of early weaning was 88% higher in children of women who consumed alcohol during pregnancy and 93% higher in those who intended to breastfeed less than six months compared to those who wished to breastfeed for six months or more. The risk of early weaning was 2.50 times higher in the children of mothers that did not practice cross-breastfeeding when compared with those who breastfed another child (Table 3).

**Table 3:** Factors associated with weaning in the first six months of life. Rio Branco, Acre, 2015–2016.

Variable		Weaning in the first six months of life	
		crude HR (95%CI)	adjusted HR^a^ (95%CI)
Breastfeeding status at discharge			
	EBF	1	1
	BF	2.24 (1.38–3.63)	1.82 (1.06–3.11)
Cross-breastfed other women’s infants			
	Yes	1	1
	No	2.46 (1.57–3.86)	2.50 (1.59–3.94)
Pacifier use			
	No	1	1
	Yes	6.28 (4.60–8.57)	6.23 (4.52–8.60)
Intended breastfeeding timing			
	> 6 months	1	1
	6 months	1.29 (0.98–1.72)	1.26 (0.93–1.70)
	< 6 months	1.80 (1.19–2.71)	1.93 (1.25–2.98)
Breastfeeding in the first hour of life			
	Yes	1	1
	No	1.38 (1.05–1.80)	1.45 (1.10–1.92)
Alcohol consumption during pregnancy			
	No	1	1
	Yes	1.62 (1.14–2.31)	1.88 (1.34–2.90)

EBF: exclusive breastfeeding; BF: breastfeeding; HR: hazard ratio.

a HR adjusted for age and maternal education, breastfeeding status at discharge, cross-breastfed other women’s infants, pacifier use, period of breastfeeding, breastfeeding in the first hour of life, alcohol consumption during pregnancy.

## DISCUSSION

Breastfeeding status at hospital discharge was one of the main factors for discontinuity of breastfeeding in the first six months of life. Children in EBF at hospital discharge were less likely to wean in six months when compared with those in BF.

Although complemented breastfeeding is not recommended, some clinical situations justify its practice^4^^,^^23^. However, complemented breastfeeding frequency found in our study was higher than that observed in Belo Horizonte, State of Minas Gerais (5.1%)^11^, similar to that of Curitiba, state of Paraná (10.2%)^14^, and lower than Australia (20.8%)^4^ and China (75%)^24^. Complemented breastfeeding may decrease the breast milk protective effect due to the loss of colostrum intestinal effec as the first feeding source. Such a loss may increase the risk of infections in early childhood^1^^,^^25^. Thus, this practice deserves special attention from health professionals directly linked to maternal and child care, and hospital managers, aiming to follow the strict criteria for offering neonates supplements during hospitalization. Thus, only children to whom the complemented breastfeeding is strictly indicated would receive it.

Hospital actions based on the *Iniciativa Hospital Amigo da Criança* (IHAC – Baby-Friendly Hospital Initiative), which aims at meeting the “Ten Steps to the breastfeeding success”^26^, must attempt to recover the practice of breastfeeding until the difficulties in the initial lactation process and BF establishment are overcome. As the initiative itself suggests, it is necessary to promote the multidisciplinary team continuing education. However, the main maternity in Rio Branco was already accredited to IHAC, while the other was still in the accreditation process as this study was conducted.

In our study, most children receiving complemented breastfeeding still in the hospital were in BF at hospital discharge (69.6%), suggesting a commitment of hospital health teams to promote exclusive breastfeeding. However, additional efforts are still needed to overcome non-clinically justified complements.

Thus, postpartum hospitalization time is important to help define the course of breastfeeding since it allows puerperal women to access professional help and receive breastfeeding awareness to increase self-confidence. Also, should be added strategic support and promotion to exclusive breastfeeding after hospital discharge^23^.

In Rio Branco, children in BF at hospital discharge had a higher risk of early weaning when compared with those in EBF at the same period. In Melbourne, Australia, infants in EBF since postpartum were more likely to continue breastfeeding for 6-month than those that received milk formula in the early postpartum period^4^. In Curitiba, Paraná, children in EBF in the maternity hospital had a longer breastfeeding duration when compared with those that received BF during postpartum hospitalization^14^. In Belgium, infants in BF at hospital discharge had a higher risk of weaning at 18 months when compared with those discharged in EBF^27^.

In this sense, the breast milk complement in the postnatal period should be seen as temporary intervention support, followed byexclusive breastfeeding encouragement. Furthermore, women at hospital discharge identified as less likely to breastfeed exclusively should receive more specific support to improve exclusive breastfeeding^15^. Thus, breastfeeding promotion actions such as the joint hospital accommodation, human milk banks, and IHAC, which aim to recover the practice of breastfeeding, especially in the hospital environment^23^^,^^26^, help increase the prevalence of breastfeeding, and EBF at hospital discharge.

The lack of breastfeeding in the first hour of life was also associated with early weaning in our study. Similar results were observed by other studies, in which late breastfeeding newborns were at a higher risk of early weaning^3^^,^^11^^,^^24^. The association between breastfeeding in the first hour of life and BF’s longer duration can be partially explained by the beneficial effect of early contact with the mother, contributing to oxytocin production and release. Such hormone is involved in milk ejection, increasing the likelihood of longer breastfeeding time^28^. An early mother-and-child contact has been described as important to improve the bonds between the two and extend breastfeeding time^28^^,^^29^.

Although not fully explored in the literature, it was interesting to find that infants whose mothers did not practice cross-breastfeeding presented 2.50-folds the risk of early weaning compared with those whose mothers practiced cross-breastfeeding. The fact that 18.6% of the mothers cross-breastfed another baby may be related to historical social, and cultural influences related to colonization inheritance in the area^6^^,^^30^. However, according to a few studies that explored this variable, cross-breastfeeding is also common in other Brazilian regions^30^^,^^31^.

Cross-breastfeeding usually occurs in physical withdrawal situations between the mother and child or when the mother reports low milk production. When the available information is insufficient to solve maternal difficulties, mothers resort to previous experiences and resources such as other foods early introduction or cross-breastfeeding^30^^,^^31^.

The milk supplied from another lactating woman, breastfeeding performed by another healthy woman, milk from a human milk bank, or substitute milk to humans are alternatives pointed out by the WHO for exceptional situations when breast milk is inadequate or unavailable^32^. The Brazilian Ministry of Health, however, formally contraindicates cross-breastfeeding under any circumstances^33^. Thus, health professionals need to evaluate the situations and contexts in which such practice occurs and discourage it.

In our study, the most strongly associated factor to early weaning was the use of pacifiers, which has been widely explored in the literature^3^^,^^7^^,^^13^. In a study with children of adolescent mothers^7^, not using pacifiers was the only factor associated with breastfeeding continuity at 6,12, and 24 months of life. In a cohort study in Cruzeiro do Sul, the state of Acre, 75% of babies that used pacifiers were no longer exclusively breastfed at the end of the first month of life, and among them, the duration of EBF was 33% shorter as compared to non-users^34^. Likewise, in a cohort study in Itaúna, state of Minas Gerais, a higher risk of BF discontinuity was observed in children who used pacifiers^3^.

The use of pacifiers is contraindicated by the WHO^26^, as the oral dynamics of breast sucking is different from pacifiers, which favors “nipple confusion” by the infant^26^, leading to early weaning^3^^,^^7^^,^^13^. The use of pacifiers may also reflect maternal difficulties, such as anxiety, insecurity, and problems in BF management^3^. Thus, the health team must provide information on the consequences of pacifier use^3^^,^^26^.

Studies show that prenatal care women that reported intention to breastfeed are more likely to start and continue breastfeeding^3^^,^^6^^,^^27^. The intention to breastfeed exclusively for six months or more substantially reduced the risk of early weaning in Hong Kong^6^. Since the intention is a precursor of the practice, in Rio Branco, the desire to breastfeed for less than six months was associated with early weaning.

Many factors experienced by a pregnant woman can affect breastfeeding plans^16^. Prenatal care opens an opportunity to guide breastfeeding, and specific professional support interventions and access to information have effectively strengthened breastfeeding. Moreover, special attention should be paid to early postpartum women since this is a risk phase for the psychological manifestation that affects the intention to breastfeed and may contribute to early weaning^16^.

Despite the adverse maternal-fetal outcomes associated with alcohol use during pregnancy^35^, this is the drug frequently used by pregnant women^35^^,^^36^. However, alcohol consumption increases the milk production antagonist hormones, reducing the ejection and the amount of breast-milk available to the infant, contributing to early weaning^37^.

A complex network of sociodemographic, behavioral, and family characteristics is associated with alcohol use during pregnancy^3^^,^^35^^,^^36^^,^^38^. Alcoholic women profile may impair BF maintenance, as observed in studies in which women who reported using alcohol during pregnancy^3^ or consumed alcohol after delivery^38^ had a higher risk of breastfeeding failure. Such results showed the importance of planned behavior in breastfeeding since women that used alcohol during pregnancy are more vulnerable to alcohol consumption during lactation, which increases the risk of early weaning^38^.

This was the first population-based study, with a prospective design, on factors associated with early weaning in the Brazilian Western Amazon. Our study is also unprecedented in characterizing the hospital breastfeeding pattern in association with weaning. Our results allowed estimating associations of certain variables which are still poorly explored in the literature, such as cross-breastfeeding, and breastfeeding pattern at hospital discharge.

Despite representing a critical limitation, the losses in our study were randomly distributed. The potential damage of such losses would be the reduction of the study power. Another limitation is the possibility of memory bias because information about breastfeeding in the first six months of life was collected when the children were between 6 and 15 months old. On the other hand, to minimize possible trends in self-reported information, breastfeeding status at the time of the interview, consumption, and age of introduction of other foods were questioned, enabling the construction of the variables related to breastfeeding. These questions avoided child misclassification into EBF, PBF, and BF categories by the mothers or answering the question based on culturally determined answers.

In conclusion, the risk of early weaning was higher among children discharged from the hospital in BF, used pacifiers, and was not breastfed in the first hour of life. Women that did not practice cross-breastfeeding intended to breastfeed for less than six months and those that consumed alcohol during pregnancy also presented a higher risk of early weaning.

Thus, considering the challenges to ensure breastfeeding during the first six months of life, our study reinforces the need to prioritize breastfeeding in the first hour of life and that encourage the child EBF until hospital discharge. Moreover, in the postpartum period, complemented breastfeeding in the hospital must occur only when clinically justified.

Studies with larger samples size exploring the risk of early weaning and changes in breastfeeding patterns among children that were discharged from the hospital in EBF are needed, considering the complemented breastfeeding in the hospital environment with and without acceptable medical justification.

Information on the contraindication of cross-breastfeeding must be included in institutional materials of the Brazilian Ministry of Health, such as The Pregnant Woman’s and Children’s Notebooks, and prenatal information and guidance on cross-breastfeeding risks, and breastfeeding adequate management should be expanded.

Health education actions should also focus on pregnancy alcohol use women, women who intend to breastfeed less than six months, and those unaware of pacifier use risks. Finally, our study should serve as a reference for health teams in Rio Branco, State of Acre, and other places with similar characteristics, on assisting pre-and postnatal care directed to risk factors associated with early weaning, since low complexity actions can cooperate for the integral promotion of children’s health from the first years of life.
