# Supplementary material for: Breastfeeding patterns and factors associated with early weaning in the Western Amazon
Source: Rev Saude Publica. 2021 May 6;55:21. doi: 10.11606/s1518-8787.2021055002134 (PMC8102026; doi:10.11606/s1518-8787.2021055002134)
Supplement: Translation

# Translation: Padrões de amamentação e fatores associados ao desmame precoce na Amazônia ocidental

**Authors:** Fernanda Andrade Martins, Alanderson Alves Ramalho, Andréia Moreira de Andrade, Simone Perufo Opitz, Rosalina Jorge Koifman, Ilce Ferreira da Silva

**Affiliations:** I Rio BrancoAC Brasil Universidade Federal do Acre. Centro de Ciências da Saúde e do Desporto. Programa de Pós-Graduação em Saúde Coletiva. Rio Branco, AC, Brasil.; II Rio de JaneiroRJ Brasil Fundação Oswaldo Cruz. Escola Nacional de Saúde Pública Sérgio Auroca. Departamento de Epidemiologia e Métodos Quantitativos em Saúde. Rio de Janeiro, RJ, Brasil.

**Keywords:** Aleitamento Materno, Desmame, Nutrição do Lactente, Saúde Infantil, Indicadores Básicos de Saúde

## Abstract

**OBJETIVO:**

Caracterizar os padrões de amamentação nos primeiros seis meses de vida e fatores associados ao desmame precoce numa coorte de nascidos vivos em Rio Branco, Acre.

**MÉTODOS:**

Estudo prospectivo com nascidos vivos entre abril e junho de 2015. As entrevistas com as mães ocorreram logo após o nascimento e entre 6 e 15 meses pós-parto. Na alta hospitalar, o aleitamento foi definido em exclusivo (AME) e materno (AM). No seguimento, os padrões de amamentação foram AME, aleitamento materno predominante (AMP) e AM. A interrupção da amamentação nos primeiros seis meses foi classificada como desmame precoce. Utilizou-se o método de Kaplan Meier (*log-rank*: 95%) para estimar a probabilidade condicional de mudança no padrão de amamentação e risco de desmame. Os fatores associados ao desmame e seus intervalos de confiança de 95% (IC95%) foram analisados pela regressão proporcional de Cox bruta e ajustada.

**RESULTADOS:**

Participaram do estudo 833 lactentes que na alta hospitalar estavam em AME (95,4%) e AM (4,6%). A probabilidade do lactente em AME na alta hospitalar permanecer em AME, ou se tornar AMP ou AM, aos seis meses, foi de 16,4%, 32,3% e 56,5% respectivamente. A probabilidade de desmame aos seis meses foi estaticamente maior para lactentes em AM na alta hospitalar (47,4%) em comparação com aqueles em AME (26%). Mostraram-se associados ao desmame precoce: o AM na alta hospitalar (HR = 1,82; IC95% 1,06–3,11), ausência de amamentação cruzada praticada pela mãe (HR = 2,50; IC95% 1,59–3,94), usar chupeta (HR = 6,23; IC95% 4,52–8,60), pretender amamentar por menos de seis meses (HR = 1,93; IC95% 1,25–2,98), não amamentar na primeira hora de vida (HR = 1,45; IC95% 1,10–1,92) e consumir álcool na gestação (HR = 1,88; IC95% 1,34–2,90).

**CONCLUSÃO:**

Comparados aos lactentes em AME, aqueles em AM, na alta hospitalar, apresentaram maior probabilidade de desmame. Esforços em saúde pública devem priorizar o AME na alta hospitalar, promover amamentação na primeira hora de vida e orientar sobre os riscos do consumo de álcool na gestação, amamentação cruzada e uso de chupeta.

## INTRODUÇÃO

A amamentação exclusiva até os seis meses de idade é um dos principais objetivos de programas de nutrição e saúde pública para reduzir a mortalidade em menores de 5 anos^1^. Os benefícios da amamentação para a saúde da criança e da mãe, a curto e longo prazo, são amplamente reconhecidos^1^, principalmente quando praticada por pelo menos dois anos e como forma exclusiva de alimentação do lactente até o sexto mês de vida, conforme recomendação da Organização Mundial da Saúde (OMS)^2^. Contudo, muitas mulheres interrompem a amamentação antes do recomendado em todo o mundo^3^^–^^7^.

Devido a ações de saúde pública empregadas nos últimos 30 anos em prol da amamentação, os inquéritos nacionais realizados a partir de 1975 retrataram aumento do aleitamento materno exclusivo (AME) em crianças entre 0 e 6 meses de vida e aumento da duração mediana da amamentação, aproximando-se das recomendações da OMS^8^. A série histórica dos indicadores de aleitamento materno (AM) no Brasil mostra tendência ascendente até 2006, com estabilização a partir dessa data nos indicadores de AME em menores de seis meses, aleitamento materno continuado com um ano de vida e aleitamento materno em menores de 2 anos, o que sugere a necessidade de avaliar e revisar as políticas e programas de promoção, proteção e apoio ao aleitamento no país^9^. Em 2008, na Pesquisa de Prevalência de Aleitamento Materno nas Capitais Brasileiras e Distrito Federal, Rio Branco, capital do estado do Acre, apresentou uma prevalência de 36,1% de AME em menores de seis meses, porcentagem inferior à média nacional no mesmo ano (41%)^10^.

Os fatores que podem levar à descontinuidade do AM incluem: primiparidade^7^^,^^11^^–^^13^, baixo peso ao nascer^3^^,^^12^^,^^14^, uso de chupeta^3^^,^^7^^,^^12^, dificuldade materna para amamentar após o parto^4^^,^^11^^,^^15^, início tardio do AM^3^, ausência de AME na maternidade^4^^,^^11^^,^^14^, conceito de tempo ideal de AM menor que seis meses^11^, desconhecimento das vantagens da amamentação^11^, falta de apoio paterno na amamentação^7^^,^^11^, trabalho materno fora do lar^12^^–^^14^, uso de drogas lícitas^3^^,^^15^, baixa idade^12^^,^^13^^,^^15^ e escolaridade materna^3^^,^^4^^,^^12^^,^^13^^,^^15^.

A intenção e a confiança em amamentar e o apoio familiar para evitar o isolamento materno no puerpério ajudam a manter a AM, enquanto a ansiedade e a inexperiência para lidar com a nova condição de ser mãe têm gerado efeito inverso^3^^,^^4^^,^^13^^,^^15^^,^^16^. Neste sentido, detectar o perfil da amamentação e fatores associados ao desmame é fundamental para elaborar políticas em prol do AM, adaptadas para cada realidade. Assim, o objetivo deste estudo foi caracterizar os padrões de amamentação nos primeiros seis meses de vida e fatores associados ao desmame precoce numa coorte de nascidos vivos em Rio Branco, Acre.

## MÉTODOS

Este estudo integra o projeto “Evolução dos indicadores nutricionais de crianças do nascimento ao primeiro ano de vida em Rio Branco, Acre”, desenhado para investigar desfechos relativos à saúde infantil de nascidos vivos (NV) nas duas únicas maternidades da capital. O estudo-matriz estimou o tamanho amostral com base no número de partos de mulheres residentes no município em 2010 (6.437)^17^. Considerando a frequência de “amamentação na primeira hora de vida” como exposição, erro tipo-I de 5%, poder de 80%, razão de expostos/não expostos de 9 e razão de risco/prevalência de 2,5, estabeleceu-se que seria necessário incluir na amostra 1.192 NV.

Todas as puérperas formalizaram sua concordância em participar do estudo por meio da assinatura de termo de consentimento livre e esclarecido. O projeto original foi aprovado pelo Comitê de Ética em Pesquisa da Universidade Federal do Acre, CAAE: 40584115.0.0000.5010.

Trata-se de estudo prospectivo em coorte de NV de Rio Branco, Acre, entre 6 de abril e 30 junho de 2015, seguidos entre o 6º e o 15º mês pós-parto. Na coorte original foram incluídos NV cujas mães residiam na área urbana de Rio Branco e não apresentavam distúrbios psiquiátricos que impossibilitassem responder a entrevista. Para o presente estudo, foram excluídos os NV gemelares, os que apresentaram contraindicação para amamentação e aqueles que não foram amamentados ou foram amamentados por menos de um dia.

Os dados foram coletados por meio de instrumentos padronizados, aplicados por uma equipe de acadêmicos da área de saúde treinados e supervisionados por pesquisadores da Universidade Federal do Acre, responsáveis pelo controle de qualidade do trabalho de campo. As entrevistas de entrada foram realizadas nas primeiras 48 horas pós-parto, e a visita de seguimento, agendada por telefone, ocorreu em domicílio. Quando o contato telefônico não foi possível, realizou-se busca ativa nos endereços previamente informados. Em caso de ausência da participante no momento da visita domiciliar, novas visitas foram feitas em horários alternados, incluindo final de semana (média de três visitas).

As variáveis independentes foram obtidas por entrevista e confirmadas diretamente no prontuário da mãe ou da criança, na declaração de nascidos vivos ou no cartão da gestante. Essas variáveis foram: idade (< 20, 20–34 ou ≥ 35 anos); cor da pele; escolaridade materna (ensino fundamental completo ou incompleto, ensino médio completo ou incompleto, ensino superior completo ou incompleto); situação conjugal (sem ou com companheiro); número de filhos vivos antes desta gestação (nenhum, 1–2 ou 3 ou mais); tabagismo na gestação (sim ou não); setor de atendimento pré-natal (público ou privado); número de consultas pré-natal (≤ 5, 6 ou > 6 consultas); tipo de parto (normal ou cesáreo); prematuridade (sim ou não); baixo peso ao nascer (sim ou não); e sexo do bebê (masculino ou feminino).

As seguintes variáveis foram obtidas exclusivamente pela entrevista na maternidade: beneficiária do Programa Bolsa Família (sim ou não); gestação planejada (sim ou não); consumo de álcool na gestação (sim ou não); amamentação na primeira hora de vida (sim ou não); complementação ao AM no hospital (sim ou não); auxílio hospitalar no manejo do AM (sim ou não); período que pretende amamentar (< 6, 6 ou > 6 meses); posse de itens (bens de consumo); grau de instrução do chefe da família (ensino fundamental completo ou incompleto, ensino médio completo ou incompleto, ensino superior completo ou incompleto).

A classe socioeconômica foi definida segundo critérios da Associação Brasileira de Empresas de Pesquisa^18^ e categorizada em classes A e B ou C, D e E. A variável “cor da pele materna” foi autodeclarada, obtida conforme a classificação do Instituto Brasileiro de Geografia e Estatística e categorizada em “branca”, “parda” e “outras”^19^. A escolaridade materna foi coletada de forma categórica, impossibilitando a análise dessa variável por ano de escolaridade. Considerou-se peso menor que 2.500 g^20^ como baixo peso ao nascer, e a prematuridade foi definida por nascimento com idade gestacional menor do que 37 semanas^21^.

Na entrevista de seguimento foram obtidas informações sobre alimentação infantil. As frequências das práticas de aleitamento materno foram estimadas na alta hospitalar e no seguimento, com base na definição da OMS^22^. As crianças que na alta hospitalar ingeriam somente leite materno foram classificadas em AME, e aquelas que recebiam leite materno e outros leites foram classificadas em AM. Durante o seguimento, o padrão de amamentação para as crianças em AME na alta foi definido em aleitamento materno predominante (AMP) quando houve o consumo de leite materno associado a água, chás ou suco de frutas, e AM quando a amamentação foi associada a outros leites ou qualquer alimento sólido ou semissólido.

O desmame foi definido como a interrupção do AM nos primeiros seis meses de vida. O tempo até o desmame foi mensurado como os dias decorridos entre a data do nascimento e a data da interrupção do AM. Os lactentes em AME, AMP ou AM aos seis meses foram censurados na coorte.

As informações que permitiram a construção da variável tempo de amamentação (dias) para cada padrão de aleitamento (AME, AMP, AM) foram: idade de introdução de água, chá ou suco (dias); idade de introdução de outros leites (dias); e idade de introdução de outros alimentos (dias). As variáveis desmame precoce (sim ou não) e tempo de aleitamento até o desmame (dias) foram construídas a partir da idade de interrupção da amamentação (dias). Também foi perguntado às mães se elas pararam de trabalhar após o nascimento do bebê (sim ou não), se praticaram amamentação cruzada (sim ou não), se os lactentes receberam leite materno de outra nutriz (sim ou não), ou se usaram chupeta (sim ou não), se a participação do pai da criança foi positiva no incentivo ao aleitamento materno (sim ou não) e quais foram os motivos de complementação hospitalar ao AM.

As características dos participantes foram descritas usando médias (desvio-padrão) para variáveis contínuas, e proporções (%) para variáveis categóricas. O teste qui-quadrado ou teste de Fisher foi utilizado para comparar as características dos participantes incluídos nas análises e aqueles perdidos no seguimento.

As probabilidades condicionais no tempo para a mudança do padrão de amamentação, aos 30, 60, 90, 120 e 180 dias, o risco de desmame segundo os padrões de AM na alta hospitalar, as variáveis sociodemográficas, os hábitos maternos, a atenção pré-natal e hospitalar e as características da criança foram estimados pelo método de Kaplan-Meier. As diferenças entre as curvas foram avaliadas pelo teste de *log-rank* (95%).

As *hazards ratios* (HR) brutas e ajustadas e seus respectivos intervalos de confiança de 95% (IC95%), entre as variáveis de exposição e o desmame, foram estimadas por meio da regressão proporcional de Cox. Na análise ajustada, considerou-se como critério de entrada no modelo um p-valor < 0,20 nas análises brutas e importância biológica em relação ao desfecho. Foram mantidas no modelo final as variáveis que apresentaram um p-valor ≤ 0,05 ou que apresentavam importância biológica. Utilizou-se o log de máxima verossimilhança para estimar os coeficientes do modelo. O R² global e parcial foi usado para avaliar a adequação do ajuste do modelo, enquanto as curvas de log menos log foram usadas para testar os pressupostos dos riscos proporcionais no tempo, sugerindo que o modelo escolhido se ajustou bem aos dados. As análises estatísticas foram efetuadas utilizando o programa *Statistical Package for the Social Sciences* versão 22.0.

## RESULTADOS

Dos 1.216 NV da coorte original, foram excluídos 52 lactentes (4,3%), por apresentarem contraindicação de amamentação (n = 5), serem gemelares (n = 22), não terem sido amamentados (n = 7) ou terem sido amamentados por menos de um dia (n = 18), restando 1.164 NV elegíveis para o presente estudo. Durante o seguimento, 331 participantes foram perdidos (28,4%) por se recusarem a responder à segunda entrevista (n = 30), por não terem sido encontrados no endereço fornecido (n = 234), por mudança de cidade (n = 44), por óbito materno (n = 2), óbito do lactente (n = 17) ou ausência de informação sobre a idade de introdução de alimentos/interrupção do aleitamento materno (n = 4). Assim, a população deste estudo foi composta por 833 lactentes, representando 71,6% dos binômios elegíveis (Figura 1). As perdas de seguimento foram comparadas aos binômios incluídos nas análises e se mostraram estatisticamente semelhantes para todas as características sociodemográficas coletadas na entrada do estudo.

**Figura 1:** Fluxograma dos participantes da coorte de nascimento “Evolução dos indicadores nutricionais de crianças do nascimento ao primeiro ano de vida em Rio Branco, Acre” elegíveis para o estudo.

As mães participantes do estudo apresentaram idade média de 25,23 anos (± 6,8, dados não apresentados), sendo que 26,3% eram menores de 20 anos, 21,7% tinham escolaridade até o ensino fundamental (completo ou incompleto), 18,8% eram beneficiárias do programa Bolsa Família, e 83,4% se autodeclararam pardas. Além disso, 69,4% compareceram a pelo menos seis consultas de pré-natal, e 85,1% fizeram o acompanhamento pré-natal no setor público. Dentre os lactentes, 51,3% eram do sexo feminino, 49% nasceram de parto cesáreo, 7,5% apresentaram baixo peso ao nascer, e 58% iniciaram a amamentação na primeira hora de vida. Na alta hospitalar, 95,4% dos lactentes estavam em AME, e 4,6% em AM. No entanto, 15% dos lactentes receberam complementação ao aleitamento materno durante a internação pós-parto (Tabela 1).

**Tabela 1:** Características dos binômios mãe-filho participantes da coorte de nascimento “Evolução dos indicadores nutricionais de crianças do nascimento ao primeiro ano de vida em Rio Branco, Acre”, 2015–2016 (n = 833).

Variável (n)^a^	Categorias	n (%)
Idade materna		
	≥ 35 anos	95 (11,4)
	20–34 anos	519 (62,3)
	< 20 anos	219 (26,3)
Cor da pele materna		
	Parda	694 (83,4)
	Branca	82 (9,9)
	Outras	56 (6,4)
Escolaridade materna^b^		
	Até o ensino fundamental	203 (24,4)
	Ensino médio	438 (52,6)
	Ensino superior	192 (23,0)
Situação conjugal		
	Com companheiro	706 (84,8)
	Sem companheiro	127 (15,2)
Trabalho materno remunerado antes da gestação		
	Sim	285 (36,0)
	Não	507 (64,0)
Parou de trabalhar por causa do nascimento do bebê		
	Não	323 (46,3)
	Sim	375 (53,7)
Classe socioeconômica		
	A e B	170 (20,6)
	C, D e E	655 (79,4)
Beneficiária do Programa Bolsa Família		
	Não	639 (81,2)
	Sim	148 (18,8)
Nº de filhos vivos antes desta gestação		
	3 ou mais	244 (29,4)
	1 ou 2	251 (30,2)
	Nenhum	335 (40,4)
Gestação planejada		
	Sim	530 (63,9)
	Não	300 (36,1)
Tabagismo na gestação		
	Não	761 (91,4)
	Sim	72 (8,6)
Consumo de álcool na gestação		
	Não	726 (87,9)
	Sim	100 (12,1)
Setor de atendimento pré-natal		
	Público	690 (85,1)
	Privado	121 (14,9)
Número de consultas pré-natal		
	≤ 5	252 (30,5)
	6	138 (16,7)
	> 6	435 (52,7)
Tipo de parto		
	Normal	425 (51,0)
	Cesáreo	408 (49,0)
Sexo da criança		
	Masculino	406 (48,7)
	Feminino	427 (51,3)
Prematuridade		
	Não	762 (92,0)
	Sim	66 (8,0)
Baixo peso ao nascer		
	Não	767 (92,5)
	Sim	62 (7,5)
Amamentação na primeira hora de vida		
	Sim	471 (58,0)
	Não	341 (42,0)
Auxílio hospitalar no manejo do aleitamento materno		
	Não	398 (48,2)
	Sim	427 (51,8)
Período que pretende amamentar		
	> 6 meses	399 (48,8)
	6 meses	339 (41,5)
	< 6 meses	79 (9,7)
Recebeu leite materno de outra nutriz		
	Não	688 (82,6)
	Sim	145 (17,4)
Amamentação cruzada praticada pela mãe		
	Sim	155 (18,6)
	Não	678 (81,4)
Uso de chupeta		
	Não	494 (59,5)
	Sim	336 (40,5)
Participação paterna positiva no aleitamento materno		
	Sim	681 (82,0)
	Não	149 (18,0)
Complementação ao aleitamento materno no hospital		
	Sim	125 (15,0)
	Não	708 (85,0)
Aleitamento materno na alta hospitalar		
	AME	795 (95,4)
	AM	38 (4,6)

AME: aleitamento materno exclusivo; AM: aleitamento materno.

a As diferenças em relação ao total são decorrentes de falta de informação na variável.

b As categorias se referem ao nível de escolaridade completo ou incompleto.

Os motivos de complementação hospitalar ao leite materno referidos pelas mães incluíram condições referentes ao lactente, como prematuridade, patologia ou hipoglicemia (54,7%), a mãe estava com pouco leite ou criança com dificuldade de sugar (35,8%), rotina hospitalar (6,3%) e uso materno de medicamento (3,2%). Trinta mães não souberam informar o motivo (dados não apresentados).

A mediana do AM foi de 180 dias, e do AME foi de 90 dias. A duração média do AM foi de 152,53 dias (± 51,25), e do AME 86,84 dias (± 62,62). A probabilidade condicional de desmame geral da amostra foi de 27% (dados não apresentados). A probabilidade de uma criança que recebeu alta hospitalar em AME permanecer em amamentação exclusiva ou tornar-se AMP ou AM aos seis meses foi de 16,4%, 32,3% e 56,5%, respectivamente. Os lactentes em AM (47,4%) na alta hospitalar apresentaram probabilidade de desmame aos seis meses maior do que aqueles em AME (26%) (Figura 2).

**Figura 2:** Padrões de aleitamento materno nos primeiros seis meses de vida (gráficos A, B e C) e risco de desmame precoce (gráfico D), segundo aleitamento materno na alta hospitalar, na coorte de nascidos vivos em Rio Branco, Acre (método de Kaplan Meier).

Os fatores estatisticamente associados ao desmame na análise bruta foram: consumo de álcool na gestação, ausência de amamentação na primeira hora de vida, auxílio no manejo do AM, pretensão de amamentar < seis meses, AM na alta hospitalar, ausência de amamentação cruzada praticada pela mãe, uso de chupeta e ausência de participação paterna positiva no AM (Tabela 2).

**Tabela 2:** Desmame nos primeiros seis meses de vida, segundo as variáveis sociodemográficas, hábitos maternos na gestação, atenção pré-natal e hospitalar e características da criança. Rio Branco, Acre, 2015–2016.

Variável		Análise de Kaplan-Meier		*Hazard ratio* (HR)
		1-Sobrevida (%)	*log-rank* (p-valor)	HR bruta (IC95%)
Idade materna			0,225	
	≥ 35 anos	22,1		1
	20–34 anos	28,9		1,19 (0,72–1,97)
	< 20 anos	24,7		1,41(0,90–2,22)
Cor da pele materna			0,285	
	Parda	26,1		1
	Branca	34,1		1,36 (0,91–2,03)
	Outras	28,6		1,13 (0,68–1,89)
Escolaridade materna^a^			0,134	
	Até o ensino fundamental	21,7		1
	Ensino médio	29,7		1,40 (1,00–1,97)
	Ensino superior	26,6		1,23 (0,82–1,84)
Situação conjugal			0,307	
	Com companheiro	26,5		1
	Sem companheiro	29,9		1,20 (0,84–1,70)
Trabalho materno remunerado antes da gestação			0,417	
	Sim	28,8		1
	Não	25,6		0,90 (0,68–1,18)
Parou de trabalhar por causa do nascimento do bebê			0,198	
	Não	23,8		1
	Sim	28,5		1,21 (0,90–1,62)
Classe socioeconômica			0,694	
	A e B	28,2		1
	C, D e E	26,6		0,94 (0,69–1,30)
Beneficiária do programa Bolsa Família			0,827	
	Não	28,0		1
	Sim	26,4		0,97 (0,70–1,37)
Nº de filhos vivos antes desta gestação			0,180	
	3 ou mais	23,0		1
	1 ou 2	27,5		1,23 (0,87–1,75)
	Nenhum	29,6		1,35 (0,98–1,88)
Gestação planejada			0,063	
	Sim	23,9		1
	Não	29,1		1,30 (0,98–1,72)
Tabagismo na gestação			0,054	
	Não	26,3		1
	Sim	34,7		1,50 (0,99–2,26)
Consumo de álcool na gestação			0,005	
	Não	25,6		1
	Sim	37,0		1,62 (1,14–2,31)
Setor de atendimento pré-natal			0,232	
	Público	25,7		1
	Privado	31,4		1,23 (0,87–1,75)
Número de consultas pré-natal				
	≤ 5	25,8	0,958	1
	6	26,8		0,05 (0,70–1,57)
	> 6	27,6		1,04 (0,77–1,40)
Tipo de parto			0,406	
	Normal	25,6		1
	Cesáreo	28,4		0,90 (0,70–1,16)
Prematuridade			0,454	
	Não	27,4		1
	Sim	22,7		0,82 (0,48–1,39)
Baixo peso ao nascer			0,109	
	Não	26,3		1
	Sim	35,5		1,42 (0,91–2,20)
Amamentação na primeira hora de vida			0,016	
	Sim	23,8		1
	Não	31,1		1,38 (1,05–1,80)
Auxílio hospitalar no manejo do aleitamento materno			0,027	
	Não	23,9		1
	Sim	30,2		1,34 (1,03–1,75)
Período que pretende amamentar			0,012	
	> 6 meses	22,8		1
	6 meses	28,6		1,29 (0,98–1,72)
	< 6 meses	38,0		1,80 (1,19–2,71)
Aleitamento materno na alta hospitalar			0,001	
	AME	26,0		1
	AM	47,4		2,24 (1,38–3,63)
Recebeu leite materno de outra nutriz			0,078	
	Não	28,3		1
	Sim	20,7		0,71 (0,48–1,05)
Amamentação cruzada praticada pela mãe			< 0,001	
	Sim	13,5		1
	Não	30,1		2,46 (1,57–3,86)
Uso de chupeta			< 0,001	
	Não	10,1		1
	Sim	51,2		6,28 (4,60–8,57)
Participação paterna positiva no aleitamento materno			0,009	
	Sim	25,3		1
	Não	34,2		1,50 (1,10–2,05)

a As categorias se referem ao nível de escolaridade completo ou incompleto.

Na análise múltipla, foi observado que o AM na alta hospitalar aumentou em 82% o risco de desmame, em comparação com o AME. Esse risco também foi 45% maior nos lactentes que não foram amamentados na primeira hora de vida. Os lactentes que usaram chupeta apresentaram um risco de desmame precoce 6,23 vezes maior do que aqueles que não usaram chupeta. O risco de desmame foi 88% maior nos filhos das mulheres que consumiram bebida alcoólica na gestação, e 93% maior naquelas que pretendiam amamentar por menos de seis meses, em comparação com aquelas que desejavam amamentar por seis meses ou mais. O risco de desmame foi 2,50 vezes maior nos filhos de mães que não praticaram amamentação cruzada, em comparação com aquelas que amamentaram outra criança (Tabela 3).

**Tabela 3:** Fatores associados ao desmame nos primeiros seis meses de vida. Rio Branco, Acre, 2015–2016.

Variável		Desmame nos primeiros seis meses de vida	
		HR (IC95%)	HR ajustada^a^ (IC95%)
Aleitamento materno na alta hospitalar			
	AME	1	1
	AM	2,24 (1,38–3,63)	1,82 (1,06–3,11)
Amamentação cruzada praticada pela mãe			
	Sim	1	1
	Não	2,46 (1,57–3,86)	2,50 (1,59–3,94)
Uso de chupeta			
	Não	1	1
	Sim	6,28 (4,60–8,57)	6,23 (4,52–8,60)
Período que pretende amamentar			
	> 6 meses	1	1
	6 meses	1,29 (0,98–1,72)	1,26 (0,93–1,70)
	< 6 meses	1,80 (1,19–2,71)	1,93 (1,25–2,98)
Amamentação na primeira hora de vida			
	Sim	1	1
	Não	1,38 (1,05–1,80)	1,45 (1,10–1,92)
Consumo de álcool na gestação			
	Não	1	1
	Sim	1,62 (1,14–2,31)	1,88 (1,34–2,90)

AME: aleitamento materno exclusivo; AM: aleitamento materno; HR: *hazard ratio*.

a HR ajustada por idade e escolaridade materna, aleitamento materno na alta hospitalar, amamentação cruzada praticada pela mãe, uso de chupeta, período que pretende amamentar, amamentação na primeira hora de vida, consumo de álcool na gestação.

## DISCUSSÃO

O status de amamentação no momento da alta hospitalar se mostrou um dos principais fatores para a descontinuidade da amamentação nos seis primeiros meses de vida. Crianças em AME na alta hospitalar apresentaram menor probabilidade de desmame aos seis meses, quando comparadas àquelas em AM.

Embora a complementação do AM não seja recomendada, há situações clínicas que a justificam^4^^,^^23^. A frequência de tal complementação encontrada em nosso estudo foi superior à observada em Belo Horizonte, Minas Gerais (5,1%)^11^, semelhante à de Curitiba, Paraná (10,2%)^14^ e inferior à da Austrália (20,8%)^4^ e da China (75%)^24^. A complementação ao AM pode diminuir o efeito protetor do leite materno, devido à perda do efeito intestinal do colostro como primeira alimentação, aumentando o risco de infecções no início da infância^1^^,^^25^. Assim, essa prática merece atenção especial dos profissionais diretamente ligados aos cuidados materno-infantis e gestores das instituições hospitalares, no sentido de respeitar os critérios rigorosos para oferta de complementos alimentares aos neonatos no período de internação, de modo que apenas as crianças a quem a complementação é estritamente indicada venham a recebê-la.

São válidas as ações hospitalares baseadas na Iniciativa Hospital Amigo da Criança (IHAC), que visa atender os “Dez passos para o sucesso do aleitamento materno”^26^ na tentativa de recuperar a prática da amamentação até que as dificuldades no processo inicial de lactação e estabelecimento do AME sejam superados. E, como a própria iniciativa sugere, é necessário promover a capacitação periódica da equipe multidisciplinar. No entanto, ressalta-se que, das duas maternidades de Rio Branco, a principal já era credenciada à IHAC, mas a outra ainda estava em processo de credenciamento no momento da pesquisa.

No presente estudo, a maioria das crianças que recebeu complementação ao AM na maternidade estavam em AME na alta hospitalar (69,6%), o que sugere um empenho das equipes de saúde hospitalar para recuperar a amamentação exclusiva. Contudo, ainda seria necessário um esforço adicional para superar a complementação por motivos que não sejam clínicos.

Assim, o período de internação pós-parto é importante para ajudar a definir o curso da amamentação, pois permite às puérperas acessar ajuda profissional e receber conselhos sobre os riscos de não amamentar, de modo a aumentar a autoconfiança. A esse aconselhamento devem ser agregadas estratégias de apoio e promoção à amamentação exclusiva também após a alta hospitalar^23^.

Em Rio Branco, as crianças em AM na alta hospitalar apresentaram maior risco de desmame precoce quando comparadas àquelas em AME no mesmo momento. Em Melbourne, Austrália, os lactentes exclusivamente amamentados desde o pós-parto tiveram maior chance de continuar a amamentação até o sexto mês do que aqueles que receberam fórmula láctea no período do pós-parto precoce^4^. Em Curitiba, Paraná, também foi observado que as crianças que receberam AME na maternidade apresentaram maior duração do aleitamento materno comparadas com aquelas que receberam AM na internação pós-parto^14^. Na Bélgica, observou-se que os lactentes em AM na alta hospitalar apresentaram maior risco de desmame aos 18 meses, comparados com aqueles que receberam alta em AME^27^.

Neste sentido, a complementação ao leite materno no período pós-natal deve ser vista como uma intervenção temporária, como apoio e encorajamento para a amamentação exclusiva. E, no caso de mulheres identificadas na alta hospitalar como menos propensas a amamentar exclusivamente, deve-se oferecer um suporte mais direcionado^15^. Desta forma, ações como o sistema de alojamento conjunto, os bancos de leite humano (BLH) e a IHAC, que visam recuperar a prática da amamentação, em especial no âmbito hospitalar^23^^,^^26^, ajudam a aumentar a prevalência da amamentação e do AME na alta hospitalar.

A ausência da amamentação na primeira hora de vida foi outro fator que se mostrou associado ao desmame nesta pesquisa. Resultados semelhantes foram observados por outros estudos, em que recém-nascidos que iniciaram a amamentação tardiamente apresentaram maior risco de desmame precoce^3^^,^^11^^,^^24^. A associação entre a amamentação na primeira hora de vida e a maior duração do AM pode ser parcialmente explicada pelo efeito benéfico do contato precoce com a mãe, contribuindo para liberação de ocitocina, o hormônio que atua na ejeção do leite, aumentando a probabilidade de maior tempo de amamentação^28^. Ainda, o contato precoce entre mãe e filho tem sido descrito como importante para fortalecer o vínculo afetivo, podendo ser responsável por maior tempo de amamentação^28^^,^^29^.

Um achado interessante e ainda pouco explorado na literatura foi o fato de que os lactentes cujas mães não praticaram a amamentação cruzada apresentaram um risco 2,50 vezes maior de desmame precoce, quando comparados àqueles cujas mães praticaram a amamentação cruzada. Essa prática, observada em 18,6% das mães deste estudo, pode se dever à influência de questões históricas, sociais e culturais que remetem à colonização da região^6^^,^^30^, embora a amamentação cruzada também seja comum em outras localidades do Brasil, segundo os poucos estudos que a exploraram^30^^,^^31^.

A amamentação cruzada geralmente ocorre em situações de afastamento físico entre a mãe e a criança, ou quando a mãe relata baixa produção de leite. Diante de situações em que as informações disponíveis são insuficientes para solucionar as dificuldades maternas, as mães recorrem a experiências pregressas e a recursos como introdução precoce de outros alimentos ou amamentação cruzada^30^^,^^31^.

A oferta de leite ordenhado de outra lactante, a amamentação realizada por uma outra mulher saudável, o leite proveniente de banco de leite humano ou o leite substituto ao humano são alternativas apontadas pela OMS para situações excepcionais, quando o leite materno é inadequado ou indisponível^32^. O Ministério da Saúde brasileiro, contudo, formalmente contraindica a amamentação cruzada, sob quaisquer circunstâncias^33^. Desta forma, os profissionais precisam avaliar as situações e contextos em que tal prática ocorre e, por meio de aconselhamento, desestimulá-la.

Neste estudo, o fator mais fortemente associado ao desmame precoce foi o uso da chupeta, que tem sido largamente explorado na literatura^3^^,^^7^^,^^13^. Em um estudo com crianças de mães adolescentes^7^, não usar chupeta foi o único fator associado à manutenção da amamentação, tanto aos 6 como aos 12 e 24 meses de vida. Em um estudo de coorte em Cruzeiro do Sul, Acre, três quartos dos bebês que usavam chupeta não eram mais amamentados exclusivamente no final do primeiro mês de vida e, entre eles, a duração do AME foi 33% menor^34^. Do mesmo modo, em um estudo de coorte em Itaúna, Minas Gerais, foi observado maior risco de descontinuidade do AM em crianças que utilizaram chupeta^3^.

O uso de chupetas é contraindicado pela OMS^26^. A dinâmica oral da sucção do seio materno é diferente da chupeta, o que favorece a “confusão de bicos” para o lactente^26^, contribuindo para a interrupção precoce do aleitamento materno^3^^,^^7^^,^^13^. O uso de chupetas pode refletir também dificuldades maternas, como ansiedade, insegurança e problemas no manejo do AM^3^. Desse modo, é importante que a equipe de saúde forneça informações sobre as consequências do uso de chupeta^3^^,^^26^.

Estudos mostram que mulheres que no pré-natal relatam intenção de amamentar são mais propensas a iniciar e continuar o AM^3^^,^^6^^,^^27^. A intenção de amamentar exclusivamente e amamentar por seis meses ou mais reduziu substancialmente o risco de interrupção da amamentação em Hong Kong^6^. Como a intenção é precursora da prática, em Rio Branco o desejo de amamentar por período menor que seis meses se mostrou associado ao desmame precoce.

Muitos fatores vividos por uma gestante podem afetar os planos de amamentar^16^. O pré-natal é um período oportuno para fornecer orientações sobre o aleitamento materno, e intervenções específicas de apoio profissional e acesso à informação têm se mostrado efetivas no fortalecimento da amamentação. Além disso, é preciso ter especial atenção às mães no período pós-parto, visto que esta é uma fase de risco para a manifestação de fatores psicológicos que afetam a intenção de amamentar e podem contribuir para o fracasso do AM^16^.

Apesar dos desfechos maternos-fetais negativos associados ao uso de álcool durante a gestação^35^, essa é a droga de abuso mais comumente utilizada pelas gestantes^35^^,^^36^. Seu consumo eleva o teor de hormônios antagonistas da produção láctea, reduzindo a ejeção e a quantidade de leite dispensada ao lactente, o que pode contribuir para o desmame^37^.

Uma rede complexa de variáveis sociodemográficas, comportamentais e familiares é associada ao uso de álcool na gestação^3^^,^^35^^,^^36^^,^^38^, e o perfil da mulher etilista pode prejudicar a manutenção do AM, como observado em estudos onde mulheres que relataram fazer uso de bebida alcoólica na gestação^3^ ou que consumiram bebida alcoólica após o parto^38^ apresentaram maior cessação do AM. Estes dados retratam a importância do comportamento planejado na amamentação, uma vez que mulheres que fizeram uso de álcool durante a gestação são mais vulneráveis ao consumo de álcool no período da lactação, o que potencializa o risco de desmame^38^.

Este foi o primeiro estudo de base populacional, com delineamento prospectivo, sobre fatores associados ao desmame nos primeiros seis meses de vida na Amazônia ocidental brasileira. Este trabalho é inédito ainda em caracterizar o padrão de amamentação hospitalar em associação com o desmame. Seus resultados permitiram estimar associações de variáveis pouco exploradas ao desmame precoce, como as variáveis amamentação cruzada e aleitamento materno na alta hospitalar.

Embora o estudo tenha sofrido perdas que significaram importante limitação, essas foram aleatórias. O prejuízo potencial de tais perdas seria a redução do poder do estudo. Outra limitação é a possibilidade de viés de memória, pois as informações sobre o aleitamento materno nos primeiros seis meses de vida foram coletadas quando as crianças tinham entre 6 e 15 meses. Por outro lado, para minimizar possíveis vieses das informações autorreferidas, foram questionados o status de aleitamento materno no momento da entrevista, consumo e idade de introdução de outros alimentos, possibilitando a construção das variáveis referentes ao aleitamento. Essas perguntas evitaram que as mães classificassem erroneamente a criança em AME, AMP ou AM, ou que respondessem ao questionamento com base em respostas culturalmente determinadas.

Em conclusão, o risco de desmame nos primeiros seis meses de vida foi maior entre crianças que receberam alta hospitalar em AM, que utilizaram chupeta e que não foram amamentadas na primeira hora de vida. As mulheres que não praticaram amamentação cruzada, que pretendiam amamentar por tempo menor que seis meses, e as que consumiram álcool durante a gestação também apresentaram maior risco de desmame precoce.

Assim, considerando os desafios para garantir o aleitamento materno nos primeiros seis meses de vida, este trabalho reforça a necessidade de que a amamentação na primeira hora de vida seja priorizada e que os profissionais de saúde ajam de modo a estimular que o lactente receba alta em AME. Ademais, é preciso que, no pós-parto, no hospital, a complementação ao AM ocorra somente quando clinicamente justificada.

São necessários estudos futuros, com amostras maiores, que explorem o risco de desmame precoce e mudanças no padrão de amamentação entre crianças que receberam alta hospitalar em AME, considerando a complementação ao AM no ambiente hospitalar com e sem justificativa médica aceitável.

Recomenda-se incluir informações sobre a contraindicação do aleitamento cruzado em materiais institucionais do Ministério da Saúde, como Cadernetas da Gestante e da Criança, bem como ampliar as informações e orientações no pré-natal acerca dos riscos da amamentação cruzada e adequado manejo do aleitamento materno.

As ações de educação em saúde também devem focar mulheres que consomem bebida alcóolica durante a gestação, que pretendem amamentar por período menor que seis meses ou que desconhecem o risco do uso de chupeta.

Por fim, espera-se que este estudo sirva de referência para equipes de saúde de Rio Branco e outros locais com características semelhantes, auxiliando a atenção pré e pós-natal direcionada a fatores de risco associados ao desmame precoce, uma vez que ações de baixa complexidade podem cooperar para a promoção integral da saúde da criança desde os primeiros anos de vida.
